# Social Work Training and Readiness for Trauma-Informed Care in Nursing Homes

**DOI:** 10.1093/geroni/igaa057.2535

**Published:** 2020-12-16

**Authors:** Nancy Kusmaul, Todd Becker

**Affiliations:** 1 UMBC, Baltimore, Maryland, United States; 2 University of Maryland, Baltimore, Baltimore, Maryland, United States

## Abstract

Most adults have experienced traumatic events (SAMHSA, 2017). Late-life traumas may compound upon trauma histories (Maschi, et al., 2013), accentuating the risks confronting older adults. Per CMS’ updated Requirements for Participation, nursing homes (NHs) must implement trauma-informed care (TIC) approaches, effective November 2019. Many NHs do not staff Masters of Social Work (MSWs), despite their expertise in providing mental health care. Notwithstanding, employed MSWs feel unprepared to help their NHs implement TIC. This presentation discusses findings from a national survey of NH social service directors (N = 932). Results showed 71% (n = 650) reported moderate to strong interest in TIC training. A Kruskal-Wallis H test revealed a statistically significant difference in TIC training interest χ2(1) = 43.690, p < .001, such that MSWs reported higher interest (M = 486.47) than non-MSWs (M = 375.23). There was no difference between those with and without a Bachelor of Social Work.

